# A three-plasma miRNA panel predicts the risk of colorectal cancer: a community-based nested case‒control study

**DOI:** 10.1038/s41598-023-31449-3

**Published:** 2023-03-14

**Authors:** Jia Liu, Binglin Chen, Man Yang, Yun Qian, Qian Shen, Hai Chen, Yunqiu Dong, Lu Wang, Jiandong Jiao

**Affiliations:** 1 Department of Epidemiology and Biostatistics, School of Public Health, Nanjing Medical University, Nanjing, Jiangsu China; 2 Department of Health Promotion & Chronic Non-Communicable Disease Control, Wuxi Center for Disease Control and Prevention (The Affiliated Wuxi Center for Disease Control and Prevention of Nanjing Medical University), Wuxi, Jiangsu China

**Keywords:** Cancer epidemiology, Cancer prevention

## Abstract

Circulating microRNAs (miRNAs) have been considered potential markers for the early detection of malignant colorectal cancer (CRC). We aimed to identify a group of miRNAs for the early detection of CRC and assess their predictive ability in a community-based population in China. A nested case‒control study consisting of 97 incident colorectal cancer cases and 103 frequency-matched healthy controls was conducted. The data were randomly assigned into a training set (60%) and a test set (40%). We selected and detected 10 kinds of miRNAs in plasma samples. Multivariate logistic regression analysis was used to identify miRNAs associated with colorectal cancer risk in the training set and test set. Then, we evaluated the predictive ability of the identified miRNAs by the receiver operating characteristic curve (ROC). In this study, three miRNAs (miRNA-29a, miRNA-125b, miRNA-145) were significantly associated with colorectal cancer risk in both the training set and test set. The sensitivity of the identified miRNAs ranged from 0.854 to 0.961. After adding the identified miRNAs, the AUC (area under the curve) value significantly increased from 0.61 to 0.71 compared with the basic model consisting of only basic demographic information. We identified a three-plasma miRNA signature that may serve as a novel non-invasive biomarker in early CRC detection and in predicting individual CRC risk in the generation population.

## Introduction

Colorectal cancer (CRC) is one of the most common malignant tumours. In 2018, the number of new cases of colorectal cancer and the number of deaths from colorectal cancer ranked third and second in the world, respectively^1,2^. Recently, with the change in lifestyle and ageing of the population, the disease burden of colorectal cancer in China has increased sharply, and the incidence and mortality in the eastern region of China are higher than those in the central and western regions^3,4^. With the improvement of diagnosis and treatment measures, the prognosis of early CRC patients has been significantly improved, and the 5-year survival rate of stage I or stage II patients has reached 91% and 82%, respectively, whereas the 5-year survival rate plummeted to 12% when the tumour progressed to stage IV^5–7^. Therefore, early identification and detection can significantly prolong the survival time of CRC patients.

MicroRNAs (miRNAs) are short single-stranded noncoding RNA composed of 19–24 nucleotides that can regulate gene expression at the posttranscriptional level^8^. They bind to the 3’ untranslated region (3’ UTR) of the target mRNA and then lead to target degradation, translation inhibition or gene silencing, which affect subsequent protein expression^9^. The abnormal expression of miRNAs is closely related to the occurrence and development of various human diseases, including cancer^10^. MiRNAs are stable in plasma and can be used as potential biomarkers for human disease^11,12^. Therefore, the identification of miRNAs as biomarkers for the early detection of diseases contributes to a better understanding of the pathogenesis of diseases and is expected to become a target for treatment.

Many studies have confirmed that miRNAs have unique advantages in tumour diagnosis, and they are one of the focuses of CRC biomarker research^13–15^. For example, Carter et al. performed a systematic review and meta-analysis and found that the sensitivity and specificity of identified miRNA reached nearly 80%^13^. However, with increased research, the results of miRNA markers in different institutions have become inconsistent. In addition, blood circulating miRNA results may differ from those in tissue. On the other hand, previous studies on miRNAs and CRC were mainly conducted in hospital-based populations, which indicates that the population representation was insufficient compared with the general population^16,17^. In the present study, we first selected 10 miRNAs that have been previously reported to be dysregulated in discriminating CRC from healthy individuals in at least 2 studies as candidate miRNAs. After validating in a sample set including 97 CRC patients and 103 healthy controls from a community-based population, we aimed to evaluate the prediction ability after including the identified miRNAs along with the traditional prediction factors.

## Material and methods

### Ethics statement

All the participants signed written informed consent forms before recruitment. This study was approved by the Institutional Review Board of Wuxi Center for Disease Control and Prevention.

### Study subjects

The study participants were recruited with random cluster sampling from residents of two communities in Wuxi city, Jiangsu, China, from April to June 2007. All participants were at least 30 years old and had lived in their current residence for more than 5 years. In addition, these participants had no intention of moving elsewhere in five years. When participants agreed to take part in the survey, they visited the designated assessment centre to provide baseline information, physical measures, and biological samples. In total, 10,858 participants (response rate 90.48%) completed the baseline survey and donated peripheral blood at the same time.

Each subject was interviewed face-to-face by trained interviewers to collect general demographic, socioeconomic status, disease history and lifestyle habits (e.g., smoking, alcohol and dietary). The physical measurements included height, weight, hip circumference (HC), waist circumference (WC), pulse rate and blood pressure, which were measured using standard instruments and protocols. For the measure indicator, the average value was calculated for 2 measures. Body mass index (BMI) was calculated as weight (kg)/height (m^2^). Within a few weeks of the baseline survey, a quality control survey involving ~ 5% of randomly selected participants was conducted to repeat the questionnaire and measure indicators. In the present study, participants were defined as smokers if they had smoked at an average of one cigarette or more per day and for at least 1 year in their lifetime, and those who drank alcohol once or more per day and for at least 1 year were defined as drinkers. In addition, central obesity was defined as waist circumference ≥ 90 cm for males or ≥ 85 cm for females.

For each participant, a 5-mL venous blood sample (fasting for more than 8 h) was collected into one procoagulant tube. The samples were then kept in a 0–4 °C refrigerator at the assessment centre and transported within 6 h to a central laboratory at the Wuxi Center for Disease Control and Prevention, where laboratory tests according to the international quality standard ISO/IEC 17025 were performed. Triglyceride (TG), total cholesterol (TC) and high-density lipoprotein cholesterol (HDL-C) levels were measured using a Biochemistry Autoanalyzer (Olympus C2734-Au640). Another 5-mL venous blood sample was collected into one EDTA tube. After centrifugation and aliquoting, the plasma cryogenic vials were stored in − 80 °C freezer, and the white and red cells were stored at − 20 °C.

### Outcome ascertainment

Follow-up was conducted chiefly through linkages to available national and local datasets. Mortality data were obtained from the national disease surveillance (DSP) system, which was published by the Chinese Center for Disease Control and Prevention. All medical certification information regarding the resident's death for each case was registered in the DSP system^18^. Information about incident cancers was collected through an established cancer registry in the study area and hospital admission system. Once the participants suffered from cancer for the first time, the demographics and ICD-coded diagnosis information could be obtained from the cancer registry system, and the hospital admission system recorded details of all hospitalized events, tumour staging and treatment procedures. Of the participants, 90.22% had at least one medical record in the hospital admission system since the baseline survey. Linkage to the cancer registry and hospital admission system was renewed annually, and they complemented each other to provide the cancer incidence status.

We defined incident CRC using the ICD-10 codes C18, C19 and C20. Each new cancer case was diagnosed pathologically after colonoscopy examination. Suspicious diagnoses were further confirmed via active follow-up by the assessment centre or by contacting the participants directly. Up to Dec 2019, 97 participants developed incident CRC. Controls were randomly selected from participants who were free of cancer at baseline and had not developed cancer. Controls were matched for age (± 5 years) and sex to incident cases.

### Candidate miRNA selection

We selected miRNAs that had been reported to be dysregulated in the comparison of CRC and healthy controls based on the following criteria: (1) at least 2 studies reported the same dysregulation directions (upregulation or downregulation) (N = 12); (2) consistent dysregulation directions in serum/plasma and tissue miRNAs were shown (N = 10); and (3) the miRNA-CRC association data were verified in the human microRNA disease database (HMDD, http://www.cuilab.cn/hmdd)^19^ (N = 10). As a result, 10 significantly altered miRNAs (miRNA-19a, miRNA-20a, miRNA-21, miRNA-24, miRNA-29a, miRNA-29b, miRNA-92, miRNA-106a, miRNA-125b, miRNA-145) were identified as candidates for further testing.

### RNA isolation and expression analysis

Total RNA was extracted from plasma using Trizol LS Reagent (Invitrogen, Carlsbad, CA) according to the manufacturer’s protocols. To control the variability in the extractions of RNA from the plasma samples, miR-16 was added to each sample. RNA was purified using the mirVana™ miRNA Isolation Kit (AM1561) according to the manufacturer’s instructions.qRT‒PCR was performed using a TaqMan microRNA probe (Applied Biosystems). Reverse transcriptase reactions were conducted with a TaqMan miRNA RT kit and stem‒loop RT primers using an ABI 7900 real-time PCR system. The reaction was carried out on a 384-well plate at 95 °C for 10 min, followed by 40 cycles at 95 °C for 15 s and 60 °C for 1 min. All reactions were performed in triplicate. The internal control used in the test was miRNA-16. The CT values were determined using the fixed threshold settings. The coefficient of variation of the CT values of triplicate samples ranges from 0.0001 to 0.088, indicating that the test results were stable. The relative expression level of each miRNA in the sample was calculated by 2^−Δ CT^. For quality control, CRC patient and control samples were randomly distributed in the well. Technicians who undertook the assays were blinded to the subjects’ case or control status.

### Statistical analysis

The distributions of continuous variables (age, BMI, WC, HC and lipid levels) were described using the mean ± standard deviation (sd). Categorical variables (sex, smoking status and alcohol consumption status) were defined as counts. The associations between miRNAs and CRC risk were calculated by logistic regression after adjusting for age, sex, smoking status, alcohol consumption and central obesity status. The analysis was conducted in the training set (60%) and validated in the test set (40%). All *P* values were two-sided, and *P* < 0.05 was considered significant. Receiver operating characteristic (ROC) curves were generated, and the area under the ROC curve (AUC) was computed to assess the discriminating effect of candidate miRNA biomarkers and traditional prediction factors. All of the statistical analyses were performed with R software (Version 2.15.3; The R Foundation for Statistical Computing, http://www.cran.r-project.org/).

## Results

The demographic and clinical characteristics of the study population are summarized in Table 1. No significant differences in demographic information (sex, age, smoking, drinking), obesity index (BMI, WC, HC) or blood lipid index (TC, TG, HDLC) between incident colorectal cancer patients and matched controls were observed. Among them, the index of obesity in the case group was slightly higher than that in the control group, but the difference was not statistically significant; in particular, the central obesity rate in the case group was marginally significantly higher than that in the control group (*P* = 0.053).

**Table 1:** Baseline characteristics of new colorectal cancer cases and healthy controls.

Variables	Case (N = 97)	Control (N = 103)	*P*
Sex			
Male	48	55	0.671
Female	49	48	
Age	63.6 ± 10.96	64.27 ± 10.72	0.661
Smoke			
Ever	21	27	0.510
Never	75	76	
Drink			
Ever	11	10	0.818
Never	85	93	
FPG (mmol/L)	5.18 ± 1.61	4.81 ± 0.91	0.048
TC (mmol/L)	4.92 ± 0.85	4.85 ± 0.74	0.545
TG (mmol/L)	2.40 ± 1.94	2.24 ± 1.70	0.535
HDLC (mmol/L)	1.38 ± 0.39	1.41 ± 0.38	0.552
BMI (kg/m^2^)	24.10 ± 3.45	23.74 ± 3.31	0.443
Waist circumference (cm)	87.22 ± 10.15	85.55 ± 10.04	0.243
Central obesity			
Yes	87	82	0.053
No	10	21	
Hip circumference (cm)	96.42 ± 7.89	94.47 ± 8.85	0.102

The original data were randomly assigned to training sets (60%) and test sets (40%), and the expression of miRNA-19a, miRNA-20a, miRNA-21, miRNA-24, miRNA-29a, miRNA-29b, miRNA-92, miRNA-106a, miRNA-125b and miRNA-145 was measured in the training sets and test sets. As shown in Table 2, after adjusting for age, sex, smoking, drinking and central obesity status, logistic regression analysis showed that miRNA-29a, miRNA-125b and miRNA-145 were significantly associated with colorectal cancer risk in both datasets (*P* < 0.05).

**Table 2:** The relationships between different miRNAs and colorectal cancer in train set and test set.

miRNA	Train set			Test set		
	OR	95%CI	*P*	OR	95%CI	*P*
miRNA-19a	1.15	0.98–1.33	0.083	0.96	0.80–1.15	0.631
miRNA-20a	1.03	1.00–1.07	0.086	1.05	0.97–1.13	0.273
miRNA-21	1.03	1.00–1.06	0.082	1.01	0.96–1.05	0.759
miRNA-24	1.00	1.00–1.01	0.576	1.00	0.99–1.02	0.584
**miRNA-29a**	**1.07**	**1.02–1.12**	**0.007**	**1.05**	**1.01–1.09**	**0.025**
miRNA-29b	1.30	1.04–1.62	0.020	1.14	0.88–1.48	0.311
miRNA-92	1.00	1.00–1.01	0.062	1.01	1.00–1.02	0.022
miRNA-106a	1.04	0.97–1.12	0.265	1.00	0.89–1.13	0.971
**miRNA-125b**	**1.34**	**1.09–1.66**	**0.006**	**1.39**	**1.02–1.88**	**0.036**
**miRNA-145**	**1.03**	**1.01–1.05**	**0.015**	**1.05**	**1.00–1.10**	**0.041**

The OR and *P* value were calculated by logistic regression after the adjustment of age, sex, smoking, drinking and central obesity status.

Significant values are in bold.

To evaluate this predictive value, we used the ROC curve to analyse the cut-off value, sensitivity and specificity of 3 kinds of positive miRNA. The cut-off value was defined as the value at which the sum of sensitivity and specificity reached its highest value. The cut-off value for miRNA-29a was 17.860 (sensitivity = 0.961, specificity = 0.316); for miRNA-125b, 2.523 (sensitivity = 0.932, specificity = 0.326); and for miRNA-145, 7.280 (sensitivity = 0.854, specificity = 0.438) (Table 3). We further evaluated the specificity and 95% CI at the specific sensitivity for the identified 3 miRNAs (Supplementary Table 1). We further evaluated the predictive value of the identified 3 miRNAs for incident colorectal cancer in different years. As shown in Supplementary Tables 2–4, from the seventh year of incidence, including the three identified miRNAs could significantly improve the predictive value of the basic model (*P* < 0.05).

**Table 3:** The cutoff value, sensitivity and specificity of 3 kinds of significant miRNAs.

miRNA	Cutoff	Sensitivity	Specificity
miRNA-29a	17.860	0.961	0.316
miRNA-125b	2.523	0.932	0.326
miRNA-145	7.280	0.854	0.438

**Figure 1:**
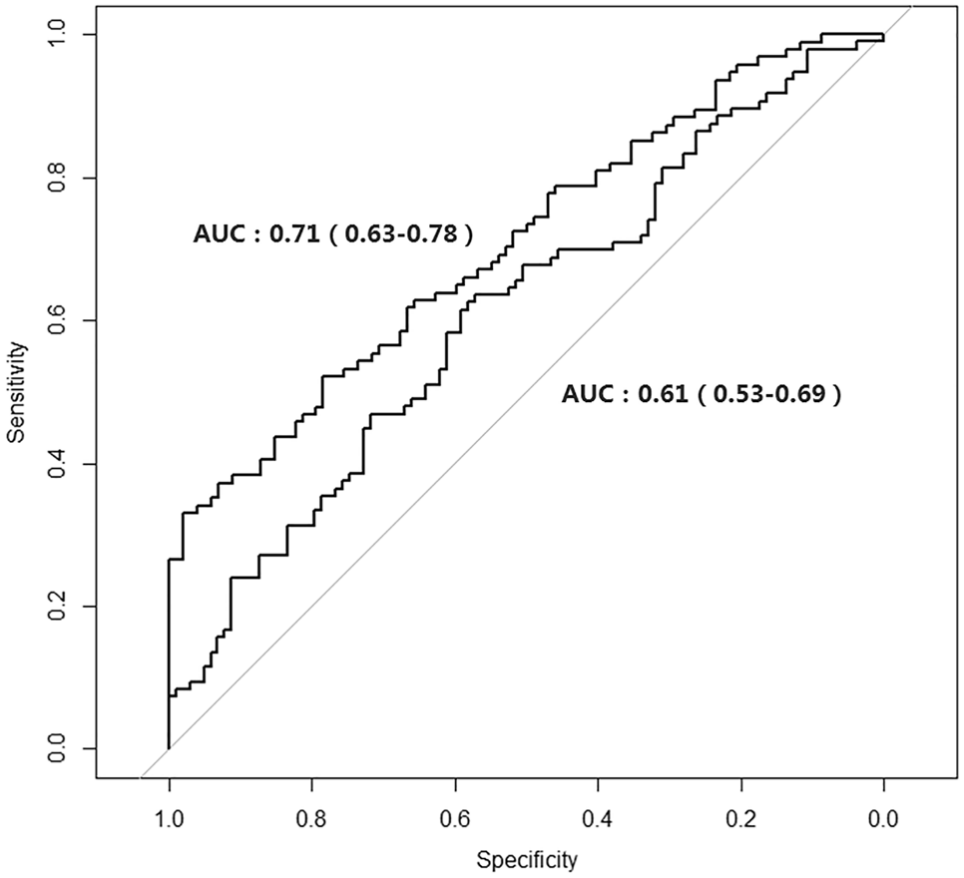
ROC curve of basic model and combined score model.

Finally, we evaluated the predictive ability of combined miRNAs along with a basic model (consisting of variables of age, sex, smoking, drinking, TC, TG, HDLC and central obesity status). The AUC value of the basic model after taking the baseline variables into account was 0.61, and the AUC value increased to 0.71 after adding the identified miRNAs (*P* = 0.009), suggesting that the model including the identified miRNAs can significantly increase the predictive efficiency of the disease. The results are shown in Fig. 1.

## Discussion

In this study, we evaluated the predictive value of selected miRNAs on incident CRC in a community-based population. Three miRNAs (miRNA 29a, miRNA125b and miRNA145) were significantly associated with incident CRC risk. The sensitivity of the identified miRNAs ranged from 0.854 to 0.961. After adding the identified miRNAs, the AUC of the basic model increased significantly. Our study provides evidence at the population level that miRNA panels might be used as early biomarkers for CRC. The case group of this study is incident CRC patients in the natural population, matched with a control group without any cancer history. The individuals in the case and control groups were enrolled in a community-based setting, indicating the high representation of participants.

For CRC diagnosis, colonoscopy is the gold standard with invasive risk^20,21^. However, colonoscopy compliance in screening programs is low in China due to positive results in primary screening, perceived severity or barriers, and limited health-care providers. The diagnostic yield was not optimal in population-based colonoscopy screening given the relatively low participation rate^22,23^. Currently, faecal-based detection methods, such as faecal immunochemical tests (FITs), are applied to identify high-risk populations before colonoscopy tests. Whereas FITs are less invasive, their specificity and ability to detect precancerous colorectal adenomas are limited^24,25^. MiRNAs are abnormally expressed in the early stages of tumour development and are deregulated in precancerous lesions and cancerous tissues. In addition, miRNAs are relatively stable in body fluids and can be easily detected in blood samples. Therefore, they can be used for monitoring for disease with minimal invasiveness^26^.

Increasing evidence indicates that dysregulated miRNA expression has a functional role in the progression and metastasis of CRC, acting either as tumour suppressors or oncogenes to regulate the expression of their specific mRNA targets. Due to their high stability, miRNAs were considered and investigated as a new class of valuable biomarkers^20^. Luo et al. conducted a screening and validation study and found that miRNA 29a was differentially expressed in CRC patients and controls^27^. In addition, studies have revealed that miRNA 29a plays a key role in colorectal cancer by regulating matrix metalloproteinase 2 and E-cadherin via KLF4^28^. In addition, miR-125b was identified to induce cetuximab resistance in CRC^29^^.^ MiR-145 mainly antagonizes SNAI1-mediated stemness and radiation resistance in colorectal cancer^30^.

At present, the diagnostic efficacy of combining multiple miRNAs is considered better than that of a single miRNA^31–33^. There are still some shortcomings in a single miRNA diagnosis. The specificity of most single miRNAs is poor, resulting in a marker often associated with a variety of diseases. Liu et al.^34^ conducted an analysis in 85 CRC patients and 78 healthy controls and found that the combination of 4 miRNAs, including miR-29a and miR-125b, showed good prediction performance (AUC = 0.952).

There were several strengths in our current study. First, participants enrolled in this study were community-based with high representation compared with hospital-based studies. Second, the selected miRNAs were verified to be associated with CRC risk in at least 2 studies to avoid inconsistent results. However, limitations in the current study should also be noted. First, the number of study participants was relatively small with limited statistical power. We will expand the study size in future studies with more new cases of CRC. Second, the predictive ability, especially specificity still be relatively low. Genotyping assay, metabolite detection and heavy metal detection are now conducted. Combined miRNA data, genome data, metabolite data and metal data together, the predictive accuracy should be improved greatly. Third, information about CRC family history and intestinal disease history was lacking. Future studies with sufficient information are warranted.

In summary, this study was carried out in a natural healthy population. The results showed that a three-miRNA signature, including miRNA-29a, miRNA-125b, and miRNA-145, could be used for the early detection of colorectal cancer. The combination of these miRNAs significantly improved the predictive ability of the basic model.

## Supplementary Information

Supplementary Tables.

## Data Availability

The datasets used and/or analysed during the current study were not freely public for sensitive information, but available from the corresponding author on reasonable request.
