# Supplementary material for: A three-plasma miRNA panel predicts the risk of colorectal cancer: a community-based nested case‒control study
Source: Sci Rep. 2023 Mar 14;13:4196. doi: 10.1038/s41598-023-31449-3 (PMC10014991; doi:10.1038/s41598-023-31449-3)
Supplement: Supplementary file 1 — Supplementary Tables. [file 41598_2023_31449_MOESM1_ESM.docx]

Supplementary Table 1 The specificity of miRNA-29a, miRNA-125b and miRNA-145

at specific sensitivity

| sensitivity | miRNA-29a | miRNA-125b | miRNA-145 |
| --- | --- | --- | --- |
| 0 | 1.00 (1.00, 1.00) | 1.00 (1.00, 1.00) | 1.00 (1.00, 1.00) |
| 0.1 | 1.00 (1.00, 1.00) | 1.00 (0.97, 1.00) | 0.97 (0.93, 1.00) |
| 0.2 | 1.00 (0.96, 1.00) | 0.97 (0.92, 1.00) | 0.95 (0.89, 0.99) |
| 0.3 | 0.95 (0.83, 1.00) | 0.93 (0.82, 0.98) | 0.92 (0.84, 0.97) |
| 0.4 | 0.84 (0.68, 0.97) | 0.84 (0.71, 0.95) | 0.86 (0.92, 0.97) |
| 0.5 | 0.73 (0.60, 0.86) | 0.74 (0.55, 0.85) | 0.76 (0.57, 0.88) |
| 0.6 | 0.64 (0.42, 0.76) | 0.60 (0.42, 0.76) | 0.60 (0.35, 0.77) |
| 0.7 | 0.45 (0.27, 0.67) | 0.47 (0.33, 0.65) | 0.39 (0.28, 0.60) |
| 0.8 | 0.27 (0.14, 0.46) | 0.37 (0.23, 0.50) | 0.31 (0.17, 0.45) |
| 0.9 | 0.11 (0, 0.24) | 0.20 (0.11, 0.37) | 0.14 (0.05, 0.29) |
| 1 | 0 (0, 0) | 0 (0, 0.16) | 0.02 (0, 0.06) |

Supplementary Table 2 The predictive value of miRNA-29a for incident colorectal cancer in different following years

| Year | Number | Median level | OR（95%CI） | *P* | AUC | | | |
| --- | --- | --- | --- | --- | --- | --- | --- | --- |
|  |  |  |  |  | mir29a | basic model | basic+mir29a model | *P* |
| Control | 103 | 1.53 | - | - | - | - | - | - |
| ≤1 | 0 | - | - | - | - | - | - | - |
| ≤2 | 2 | 1.94 | 0.97 (0.67-1.41) | 0.886 | 0.515 | 0.981 | 0.981 | - |
| ≤3 | 5 | 1.97 | 0.97 (0.82-1.15) | 0.723 | 0.521 | 0.841 | 0.854 | 0.349 |
| ≤4 | 9 | 2.19 | 1.02 (0.98-1.06) | 0.260 | 0.571 | 0.781 | 0.781 | 0.369 |
| ≤5 | 12 | 2.46 | 1.03 (1.00-1.07) | 0.070 | 0.598 | 0.683 | 0.737 | 0.169 |
| ≤6 | 11 | 3.15 | 1.04 (1.00-1.07) | 0.024 | 0.615 | 0.658 | 0.736 | 0.078 |
| ≤7 | 7 | 4.52 | 1.05 (1.01-1.08) | 0.008 | 0.652 | 0.642 | 0.753 | 0.014 |
| ≤8 | 8 | 4.52 | 1.04 (1.01-1.08) | 0.010 | 0.634 | 0.607 | 0.713 | 0.012 |
| ≤9 | 14 | 4.53 | 1.05 (1.01-1.08) | 0.006 | 0.626 | 0.579 | 0.690 | 0.006 |
| ≤10 | 11 | 5.39 | 1.06 (1.02-1.09) | 0.001 | 0.644 | 0.601 | 0.688 | 0.055 |
| ≤11 | 10 | 5.99 | 1.06 (1.02-1.09) | <0.001 | 0.664 | 0.611 | 0.693 | 0.036 |
| ≤12 | 8 | 5.39 | 1.05 (1.02-1.09) | 0.001 | 0.647 | 0.611 | 0.690 | 0.025 |

Supplementary Table 3 The predictive value of miRNA-125b for incident colorectal cancer in different following years

| Year | Number | Median level | OR（95%CI） | *P* | AUC | | | |
| --- | --- | --- | --- | --- | --- | --- | --- | --- |
|  |  |  |  |  | mir125b | basic model | basic+mir125b model | *P* |
| Control | 103 | 0.28 | - | - | - | - | - | - |
| ≤1 | 0 | - | - | - | - | - | - | - |
| ≤2 | 2 | 0.21 | 0.27 (0.01-11.81) | 0.497 | 0.558 | 0.981 | 0.981 | - |
| ≤3 | 5 | 0.33 | 0.64 (0.21-1.92) | 0.427 | 0.530 | 0.841 | 0.850 | 0.680 |
| ≤4 | 9 | 0.43 | 1.15 (0.93-1.41) | 0.197 | 0.600 | 0.781 | 0.781 | - |
| ≤5 | 12 | 0.50 | 1.21 (0.99-1.49) | 0.061 | 0.628 | 0.683 | 0.735 | 0.167 |
| ≤6 | 11 | 0.44 | 1.27 (1.05-1.52) | 0.012 | 0.636 | 0.658 | 0.729 | 0.093 |
| ≤7 | 7 | 0.67 | 1.31 (1.09-1.57) | 0.004 | 0.666 | 0.642 | 0.746 | 0.016 |
| ≤8 | 8 | 0.67 | 1.30 (1.09-1.56) | 0.004 | 0.660 | 0.607 | 0.721 | 0.006 |
| ≤9 | 14 | 0.64 | 1.30 (1.10-1.55) | 0.003 | 0.654 | 0.579 | 0.695 | 0.003 |
| ≤10 | 11 | 0.72 | 1.34 (1.12-1.59) | 0.001 | 0.666 | 0.601 | 0.699 | 0.006 |
| ≤11 | 10 | 0.73 | 1.37 (1.15-1.63) | <0.001 | 0.682 | 0.611 | 0.700 | 0.013 |
| ≤12 | 8 | 0.67 | 1.35 (1.13-1.60) | <0.001 | 0.665 | 0.611 | 0.688 | 0.017 |

Supplementary Table 4 The predictive value of miRNA-145 for incident colorectal cancer in different following years

| Year | Number | Median level | OR（95%CI） | *P* | AUC | | | |
| --- | --- | --- | --- | --- | --- | --- | --- | --- |
|  |  |  |  |  | mir145 | basic model | basic+mir145 model | *P* |
| Control | 103 | 0.78 | - | - | - | - | - | - |
| ≤1 | 0 | - | - | - | - | - | - | - |
| ≤2 | 2 | 1.55 | 0.86 (0.58-1.28) | 0.460 | 0.442 | 0.981 | 0.976 | 0.480 |
| ≤3 | 5 | 2.44 | 1.00 (0.94-1.06) | 0.981 | 0.567 | 0.841 | 0.836 | 0.759 |
| ≤4 | 9 | 1.92 | 1.02 (1.00-1.04) | 0.099 | 0.595 | 0.781 | 0.807 | 0.253 |
| ≤5 | 12 | 1.78 | 1.02 (1.00-1.04) | 0.040 | 0.596 | 0.683 | 0.748 | 0.108 |
| ≤6 | 11 | 2.11 | 1.02 (1.00-1.04) | 0.044 | 0.606 | 0.658 | 0.720 | 0.118 |
| ≤7 | 7 | 4.00 | 1.02 (1.00-1.05) | 0.028 | 0.637 | 0.642 | 0.718 | 0.039 |
| ≤8 | 8 | 4.00 | 1.02 (1.00-1.05) | 0.021 | 0.624 | 0.607 | 0.686 | 0.032 |
| ≤9 | 14 | 2.97 | 1.02 (1.00-1.04) | 0.015 | 0.621 | 0.579 | 0.664 | 0.013 |
| ≤10 | 11 | 4.39 | 1.03 (1.01-1.05) | 0.004 | 0.638 | 0.601 | 0.683 | 0.013 |
| ≤11 | 10 | 5.17 | 1.03 (1.01-1.05) | 0.003 | 0.651 | 0.611 | 0.685 | 0.020 |
| ≤12 | 8 | 4.98 | 1.03 (1.01-1.05) | 0.004 | 0.641 | 0.611 | 0.684 | 0.012 |
